# The revised Psychosis Attachment Measure: Measuring disorganized attachment

**DOI:** 10.1111/bjc.12249

**Published:** 2020-05-15

**Authors:** Catherine Pollard, Sandra Bucci, Angus MacBeth, Katherine Berry

**Affiliations:** ^1^ Division of Psychology and Mental Health Faculty of Biology, Medicine and Health School of Health Sciences Manchester Academic Health Sciences The University of Manchester UK; ^2^ School of Health in Social Science University of Edinburgh UK

**Keywords:** adult attachment, attachment, disorganized attachment, measure, Psychosis

## Abstract

**Objectives:**

The Psychosis Attachment Measure (PAM) is currently the most widely used and validated measure of attachment in psychosis. However, the PAM does not assess disorganized attachment, the type of attachment that has been most closely linked with vulnerability to psychosis. This study aimed to expand the PAM to capture the concept of disorganized attachment and to examine its psychometric properties in a psychosis sample.

**Methods:**

Clinical and academic experts in the field of psychosis and service user representatives were asked to assess the comprehensiveness and comprehensibility of the pool of disorganized items. This process resulted in 12 items hypothesized to capture disorganized attachment that were included with the original items of the PAM. A sample of 144 individuals with either a self‐reported diagnosis of, or treatment for, a psychosis‐related condition completed a battery of online measures comprising the revised PAM, existing measures of adult disorganized attachment and constructs hypothesized to be conceptually related to disorganized attachment.

**Results:**

An exploratory factor analysis was conducted with three factors retained; these were labelled anxious, avoidant and disorganized attachment. The factors displayed good internal consistency and test–retest reliability and the disorganized factor displayed good construct validity with related measures and constructs.

**Conclusions:**

These results provide preliminary evidence that the revised PAM captures the concept of disorganized attachment. However, confirmatory psychometric evaluation of the revised PAM is required, within a separate psychosis sample, to confirm its factor structure. The relationship between these results and the current literature, in addition to the clinical and research implications, are discussed.

**Practitioner points:**

- We present an expanded version of the Psychosis Attachment Measure (PAM), revised to capture the concept of disorganised attachment in adulthood. This expanded measure showed good reliability and the new disorganized subscale demonstrated construct validity.
- These results provide preliminary evidence that disorganized attachment can be measured using a simple self‐report measure with individuals with psychosis.
- Further research is required to confirm the structural dimensionality of the revised PAM within a new sample using confirmatory factor analysis.
- Following further psychometric validation the use of this measure has the potential to be expanded to other mental health conditions in which disorganized attachment has been implicated in the development and maintenance of difficulties, for example, trauma‐related conditions and borderline personality disorder.

Psychosis is a significant mental health problem, around 1 in 150 individuals will be diagnosed with a psychotic disorder at some point during their lifetime (Moreno‐Küstner, Martín, & Pastor, 2018). Psychosis is often characterized by symptoms including hallucinations, delusions, paranoia, disorganized speech and behaviour (McGrath, Saha, Chant, & Welham, 2008) as well as increased levels of interpersonal difficulties and vocational and self‐care impairments (Penn *et al.*, 2004). Theoretical models have attempted to establish the underpinnings which predispose individuals to psychosis and the mechanisms by which problems are maintained. Disruptions in attachment patterns have been found to be an important factor in the development and maintenance of psychosis.

According to Bowlby (1969), the founder of Attachment Theory, early experiences with caregivers in infancy and childhood guide interactions with others in adulthood via the development of ‘internal working models’, which are formed through interactions with early caregivers. Internal working models are mental representations of the self and expectations regarding the behaviour of others in close relationships, influencing future interpersonal functioning and methods of regulating distress (Bowlby, 1988).

In terms of attachment styles, the crucial component in determining whether infants develops a secure versus insecure attachment is the caregiver's sensitivity to the infant's distress (De Wolff & van Ijzendoorn, 1997), infants develop a secure attachment when caregivers are responsive and sensitive to distress. In adulthood, this style is associated with the ability to regulate affect and manage distress, a positive self‐image and security and autonomy in forming relationships with others. In contrast, insecure attachment is the result of suboptimal caregiving where caregivers are unresponsive or insensitive to distress. In response to this type of parenting, the infant either intensifies the level of their distress in an attempt to get their attachment needs met (insecure‐anxious attachment), which in adulthood is associated with high levels of affect and sensitivity to rejection from others, or disengages their attachment system (insecure‐avoidant attachment), which is associated with avoidance of close relationships and low levels of affect in adulthood (Shaver & Mikulincer, 2002). These attachment patterns are considered ‘organized’ as they provide coherent attempts of responding to the caregiver environment.

Main and Solomon (1986) identified a fourth ‘disorganized’ attachment style observed in infants who exhibited contradictory, disoriented and disorganized behaviours in response to reunion with caregivers. Disorganized attachment is thought to be an expression of fear due to the infant experiencing ‘fright without solution’ at being faced with the biological contradiction that their caregiver is not only their genetically programmed ‘safe haven’ but also the source of their fear. There are numerous routes to the development of disorganized attachment, including caregiver maltreatment (e.g., emotional, physical or sexual abuse), as well as more indirect but repeated insensitive parenting behaviours developing from factors such as unresolved parental trauma (van IJzendoorn, Schuengel, & Bakermans‐Kranenburg, 1999). In adulthood, disorganized attachment is partly characterized by individuals vacillating between approach and avoidance behaviours in relationships, desiring closeness with others, but fearing rejection and intimacy (Bartholomew, 1994). These approach‐avoidance behaviours have been conceptualized as fearful attachment on several self‐report measures of attachment (Bartholomew & Horowitz, 1991; Griffin & Bartholomew, 1994). The Attachment Interview's (AAI; George, Kaplan, & Main, 1996 ) unresolved classification, which is understood to correspond theoretically to infant disorganized attachment, classifies those as unresolved who appear disoriented or disorganized when discussing their attachment history. For example, individuals categorized as unresolved on the AAI may display bizarre, incomprehensible and unpredictable lapses of their narrative (Madigan *et al.*, 2006; van IJzendoorn, 1995). Fearful attachment on self‐report measures is understood to conceptually overlap with unresolved attachment on the AAI (Bartholomew, 1994).

It is now well established that trauma increases the risk of developing psychosis (Varese *et al.*, 2012). Although trauma can occur without psychosis onset, significant associations have been found between voice‐hearing and paranoia and early adverse child experiences, such as emotional, physical or sexual abuse (Bentall, Wickham, Shevlin, & Varese, 2012; Varese *et al.*, 2012), which involve threats to the development of secure attachments (Berry & Bucci, 2016). In addition, a psychological defence to trauma, dissociation, has been identified as being significantly associated with the development of voice‐hearing (Pilton, Varese, Berry, & Bucci, 2015; Varese, Barkus, & Bentall, 2011) and paranoia (Pearce *et al.*, 2017). It has been hypothesized that the quality of earlier relationships, and disorganized attachment more specifically, may offer a diathesis for dissociation (Longden, Madill, & Waterman, 2011). Attachment theory more generally has been shown to be important in understanding psychosis (Berry, Roberts, Danquah, & Davies, 2014), with studies showing associations between attachment avoidance and voice‐hearing (e.g., Berry, Barrowclough, & Wearden, 2008; Ponizovsky, Vitenberg, Baumgarten‐Katz, & Grinshpoon, 2013) and paranoia (Bentall *et al.*, 2012). In the largest study to date examining attachment profiles in psychosis, Bucci, Emsley, and Berry (2017) found that a disorganized attachment pattern was associated with a higher proportion of sexual and physical abuse and more positive symptoms, such as delusions and hallucinations, compared with other attachment patterns, suggesting disorganized attachment might be a more putative attachment pattern compared with other types of attachment for positive psychotic symptoms.

Further research is needed to further delineate the role of disorganized attachment in the development and maintenance of psychosis. This necessitates the availability of reliable, valid and practical measures of disorganized attachment. However, the concept of disorganized attachment is not currently well‐captured by self‐report measures of attachment styles (Berry, Varese, & Bucci, 2017). The Psychosis Attachment Measure (PAM; Berry et al., 2008) is the most widely used self‐report measure of attachment in psychosis (Bucci *et al.*, 2017). The PAM has demonstrated good psychometric properties in studies investigating psychotic experiences in clinical samples (Berry, Wearden, Barrowclough, & Liversidge, 2006, 2008). A limitation of the PAM is that it assesses the dimension of insecure attachment (anxious and avoidant); it does not capture the assessment of disorganized attachment.

When the PAM was developed (over 10 years ago), the concept of studying attachment styles in people experiencing psychosis was relatively novel and in designing the measure the authors were guided by the self‐report attachment literature which suggested that two dimensions of anxious and avoidant attachment underlie existing self‐report measures (Brennan, Clark, & Shaver, 1998). However, with growing acceptance of psychosocial models of psychosis, including the role of interpersonal traumas and attachment‐based experiences in the development of psychosis, there has been an increasing recognition of the potential importance of the concept of disorganized attachment in addition to the two traditional dimensions (Berry *et al.*, 2017).

While a number of self‐report measures do exist which capture aspects of disorganized attachment (e.g., the Relationship Questionnaire, RQ, Bartholomew & Horowitz, 1991; Adult Disorganised Attachment, ADA Paetzold, Steven Rholes, & Kohn, 2015), they focus on close interpersonal relationships, with some items specifically referring to romantic relationships, which may make them less relevant to individuals with psychosis who are often socially isolated (Redmond, Larkin, & Harrop, 2010; Trémeau, Antonius, Malaspina, Goff, & Javitt, 2016) and experience difficulty maintaining intimate relationships (Thornicroft, Brohan, Rose, Sartorius, & Leese, 2009; Wright, Wright, Perry, & Foote‐Ardah, 2007). In contrast, the PAM was developed to overcome this problem; items do not refer specifically to romantic relationships and can therefore be administered to individuals who are not currently, or who have not been recently, in romantic relationships.

## Study aims

The aims of this study were to:

1. revise the PAM by developing a disorganized attachment subscale;
2. determine whether participants' responses on the revised PAM load on three factors: anxious, avoidant and disorganized attachment;
3. assess the reliability of the revised PAM through adequate internal consistency and test–retest reliability within a 2‐week period;
4. determine whether the disorganized subscale displays concurrent validity with existing self‐report measures of adult disorganized attachment (RQ; Bartholomew & Horowitz, 1991 and the ADA, Paetzold *et al.*, 2015) and
5. examine whether hypothesized associations are identified between disorganized attachment and related constructs.

## Method

### Phase 1: Disorganized attachment item pool generation and refinement

The new disorganized attachment items were created as part of an iterative process involving four main stages of development: (1) a literature review and examination of existing attachment measure items; (2) reviewing representative AAI transcripts featuring narratives consistent with disorganized attachment for conceptual understanding of the disorganized attachment construct; (3) content validity examination with 23 clinical and research experts and (4) face validity examination through cognitive interviewing with two service user representatives.

A large pool of disorganized items was created on the basis of the above stages and the research team collaboratively reviewed and revised the items with the primary goal of reducing any obvious redundancy. This resulted in 30 items remaining. To assess content validity and to reduce the initial item pool, clinical and academic experts in the field were contacted via email for their opinion on the relevance, comprehensiveness and comprehensibility of the remaining items. Twenty‐three experts were asked to rate the relevance of the 30 items using a 4‐point Likert scale (1 = ‘not relevant’, 4 = ‘highly relevant’). Using the Content Validity Index (CVI; Lynn, 1986), 19 items which scored above 0.7 were retained. Four items were also added to the pool, resulting in 23 items. The 23 items were reassessed by the research team to remove further redundancy. This resulted in 12 items being retained. To further refine the items and to assess face validity, a cognitive interview (see Peterson, Peterson, & Powell, 2017) was conducted with two service user representatives with lived experience of psychosis with the revised items. Following this process, the wording of three of the items was revised.

### Phase 2: Psychometric examination of the revised PAM

#### Participants

Participants were recruited online between November 2018 and March 2019. Participants were eligible to take part if they met the following inclusion criteria: they self‐reported a diagnosis of a psychosis‐related difficulty or had received medication or treatment for experiences related to psychosis; were 18 years or older and were proficient in English.

#### Measures

*Demographics questionnaire:* this included age range, gender, ethnicity, level of education, marital status, psychiatric diagnosis and current or historic treatment for experiences related to psychosis.

*Relationship Questionnaire (RQ;* Bartholomew & Horowitz, 1991): consists of four paragraphs describing four prototypic attachment styles: secure, preoccupied, dismissing and fearful attachment. Continuous scores are assessed by asking participants to rate each of the prototypic descriptions on a 7‐point Likert scale from 1 (not at all like me) to 7 (very much like me). The RQ has been shown to have reasonable reliability and validity (Griffin & Bartholomew, 1994).

*Adult Disorganised Attachment (ADA;* Paetzold *et al.*, 2015 *):* this nine‐item unidimensional measure assesses adult disorganized attachment. Respondents are required to rate the degree to which they agree with each item on a 7‐point Likert scale from 1 (strongly disagree) to 7 (strongly agree). Internal consistency has been found to be good, α = .91 (Paetzold *et al.*, 2015), and internal consistency in this sample was very good (α = .882).

*The Brief Betrayal Trauma Survey (BBTS;* Goldberg & Freyd, 2006 *):* consists of 12 items and was used to assess exposure to interpersonal trauma (items 3–10). Participants were required to rate on a 3‐point Likert scale (never, one or two times or more than that) their experience of exposure to a range of adverse life events ‘Before 18’ and ‘After 18’. Good psychometric properties have been established for the BBTS, including test–retest reliability (Goldberg & Freyd, 2006) and construct validity (Deprince & Freyd, 2004). Internal consistency in this sample was very good (α = .809 Before 18; α = .821 After 18).

*Dissociative Experiences Scale (DES‐II;* Carlson & Putnam, 1993 *):* is a self‐report measure of amnesia, depersonalization, derealization and absorption and consists of 28 items which require participants to rate from 0 to 100% the extent to which they have experienced each item. Good psychometrics for reliability and validity have been reported (Holtgraves & Stockdale, 1997). Internal consistency in this sample was excellent (α = .958).

*The Community Assessment Psychic Experiences – 42 (CAPE;* Stefanis *et al.*, 2002 *):* a 42‐item self‐report measure assessing positive and negative psychotic symptoms and depressive symptoms. Only the positive symptom subscale was used in this study. Participants were asked to indicate the frequency of psychotic symptoms using a 4‐point Likert scale from 0 (Never) to 3 (Nearly always). The CAPE has demonstrated good psychometric properties with both clinical and non‐clinical participants (Stefanis *et al.*, 2002; Yung *et al.*, 2009). Internal consistency in this sample was excellent (α = .922).

*Revised Psychosis Attachment Measure (PAM;* Berry et al., 2008 *):* the original PAM (Berry *et al.*, 2008) consists of 16 items, eight of the items assess attachment avoidance and eight items assess attachment anxiety. These items originated from existing self‐report measures of adult attachment (Brennan *et al.*, 1998). The new 12 disorganized items were interspersed with the original PAM items, therefore, changing the order of items. The original administration of the PAM was retained; respondents were required to rate the extent to which each item represents how they relate to key people in their life on a 4‐point Likert scale from 0 (not at all) to 3 (very much). Three of the original PAM items are reverse scored, items 3, 6 and 15 in this study. The PAM has demonstrated good psychometric properties in studies investigating psychotic experiences in clinical samples (Berry et al., 2008). The PAM has exhibited good reliability with Cronbach's alpha for the anxiety subscale of .96 and the avoidance subscale of .86 (Berry et al., 2006).

#### Procedure

The University Research Ethics Committee approved all procedures. Participants were recruited online through posting on social media (Facebook, Twitter and Reddit) to advertise the study. Additionally, mental health charities and support groups were contacted asking if information regarding the study could be made available on any appropriate websites or social media. Once online informed consent had been obtained, participants were directed to the battery of questionnaires. Following completion of study questionnaires, consent was requested for participants to be re‐contacted in 2 weeks to complete the revised PAM again to assess test–retest reliability.

#### Data analysis

Data were collected and entered into IBM SPSS Statistics Version 25. Distribution of the data was assessed which revealed the majority of variables were not normally distributed. Accordingly, non‐parametric tests were used. Missing data were pro‐rated with the median for that scale as there were no incidents of more than 10% of the scale data missing.

An exploratory factor analysis (EFA) was conducted with Principal Axis Factoring extraction. Arguably we could have conducted a confirmatory analysis given we have hypotheses about which items were disorganized and previous data suggesting which items were likely to represent anxiety and avoidance subscales, however, given all items as a whole had not been subjected to a factor analysis we erred on the side of caution and conducted a more exploratory analysis at this stage of scale development. In terms of assumptions for EFA, the Kaiser‐Meyer‐Olkin (KMO; Kaiser, 1974) was calculated and Bartlett's test of sphericity was measured in order to determine whether EFA was appropriate. Items with inter‐item correlations >.30 and <.90 were retained for the analysis (Tabachnick & Fidell, 2013). The theoretical underpinnings of the measure, parallel analysis and the visual scree plot were taken into account when determining the number of factors to be extracted. In parallel analysis, eigenvalues arising from a random data set with equivalent sample size and variable numbers are compared with the observed eigenvalues from the data. Eigenvalues are retained if they are larger than the 95th percentile of the corresponding eigenvalues from the random data set. The visual scree plot involves plotting the eigenvalues on a graph. This is used to establish when decreases in successive eigenvalues become less evident and is called the ‘elbow’. Eigenvalues before the elbow are retained. On the basis that adult disorganized attachment is conceptualized to involve both approach and avoidance behaviours in relationships (Bartholomew, 1994), it was hypothesized that disorganized attachment would correlate with both attachment anxiety and avoidance. Oblique factor rotation, Direct Oblimin, was therefore explored and the correlation matrix extracted to determine correlations between factors (Field, 2009). Items with factor loadings <.4 were then removed from the factors (Hinkin, 1995, 1998) along with any items which cross‐loaded substantially on more than one factor (>.4; Costello & Osborne, 2005).

The Kruskal–Wallis test was used to determine whether there were any differences between scores on the revised PAM and demographic variables. Cronbach's alpha was calculated to assess internal consistency. To determine test–retest reliability intra‐class correlation coefficients (ICCs) were calculated between scores on the revised PAM measure at Time 1 (T1) and Time 2 (T2). To examine construct validity, Spearman's rank‐order correlations were performed.

## Results

### Sample characteristics

A summary of the sample demographic and clinical characteristics is provided in Tables 1 and2, respectively. A total of 144 participants completed the revised PAM, with 90.38% completing all the questionnaires to the end. Test–retest reliability was completed at T2 by 52 participants within an average time frame of 16.1 days (*SD* = 3.59). The only questionnaire with items missing was the BBTS; missing data were less than 10%. Participant age ranges varied from 18–24 to 65–74. The majority of participants were women and white British with a diagnosis of schizophrenia, currently receiving antipsychotic medication for delusions and currently receiving mental health support for delusions.

**Table 1:** Demographics

	*n*	%
Gender		
Female	94	63
Male	47	32
Other	8	5
Age range		
18–24	48	32
25–34	45	30
35–44	30	20
45–54	15	10
55–64	10	7
65–74	2	1
Ethnicity		
White British	68	45
White Irish	18	12
Any other white background	46	31
Mixed – White and Black Caribbean	13	9
Mixed – White and Black African	5	3
Sexual orientation		
Heterosexual or Straight	106	71
Gay or Lesbian	28	19
Bisexual	1	1
Other	3	2
Prefer not to say	1	1
First language		
English	132	88
Other	18	12
Relationship status		
Never married and never registered a same‐sex civil partnership	106	71
Married	28	19
Separated, but still legally married	1	1
Divorced	11	7
Widowed	3	2
In a registered same‐sex civil partnership	1	1
Education		
Degree‐level qualification	74	49
Teaching qualification or HNC/HND, BEC/TEC Higher, BTEC Higher or NVQ level 4	6	4
'A'Levels/SCE Higher or ONC/OND/BEC/TEC not higher or City & Guilds Advanced Final Level NVQ level 3	18	12
'O'Level passes (Grade A‐C if after 1975) or City & Guilds Craft/Ord level or GCSE (Grades A‐C) or NVQ level 2	5	3
CSE Grades 2‐5 GCE 'O'level (Grades D & E if after 1975) GCSE (Grades D, E, F, G) or NVQ level 1	5	3
CSE ungraded	1	1
Other qualifications	24	16
No qualifications	17	11
Current employment		
Employee	44	29
Self‐employed	11	7
Unemployed	20	13
Full‐time education at school, college or university	31	21
Looking after family/home	4	3
Receipt of sickness or disability benefits	35	23
Retired	3	2
Other inactive	2	1

**Table 2:** Clinical characteristics

	*n*	%
Received psychiatric diagnosis		
Yes	142	95
Diagnosis received		
Schizophrenia	53	35
Schizoaffective	36	24
Schizophreniform	3	2
Depression with psychotic features	47	31
Delusional Disorder	5	3
Bipolar disorder	34	23
Brief psychotic disorder	34	23
Any other which included psychotic experiences	31	21
Other	28	19
Currently receiving antipsychotic medication^a^		
Hallucinations	59	39
Delusions	70	47
Paranoia	62	41
Unusual beliefs	41	27
No	66	44
Currently receiving mental health support^b^		
Hallucinations	103	69
Delusions	110	73
Paranoia	103	69
Unusual beliefs	79	53
No	18	12
Been in hospital for mental health (MH) difficulties		
Yes	103	69
Are you currently in hospital for MH difficulties		
Yes	2	1
Received input from CMHT or early intervention service		
Yes	95	63
Currently receiving input from CMHT or early intervention service		
Yes	62	41

^a^ The participants were able to select as many symptoms that applied to them for which they were receiving antipsychotic medication.

^b^ Additionally, the participants were able to select one or more symptoms for which they were receiving mental health support.

### Exploratory factor analysis

Preliminary exploration of the factor structure of the PAM with the new disorganized items is described below. The overall KMO was ‘great’ at .880 (Field, 2009; Hutcheson & Sofroniou, 1999) and individual KMO ranged from .751 to .944, signifying sufficient sample size for EFA (Field, 2009). Bartlett's test of sphericity was also highly significant (*p* < .001) indicating EFA was appropriate.

**Figure 1:**
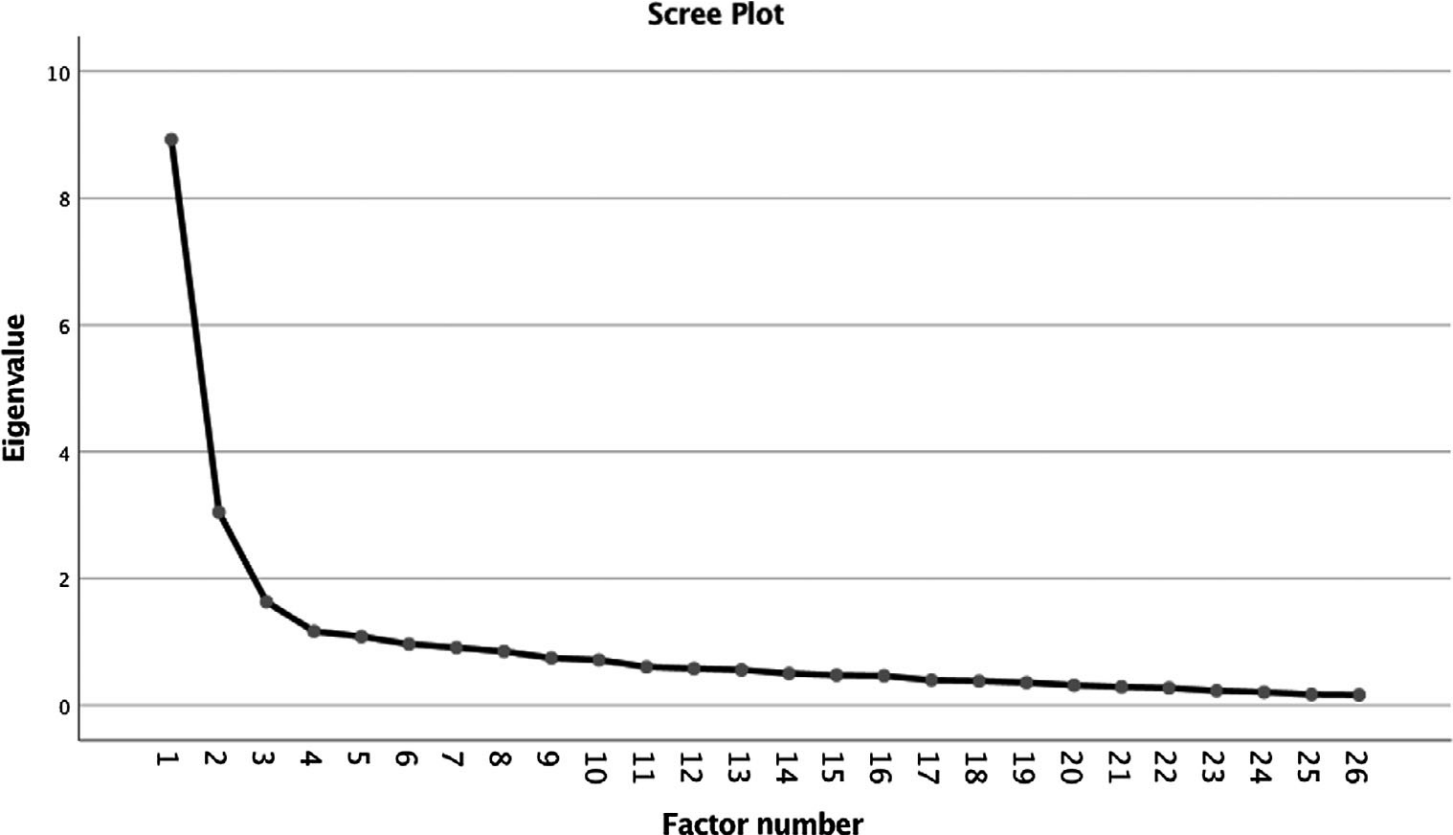
Scree plot.

On theoretical grounds, we hypothesized that the revised PAM would form three factors. Figure 1 shows the scree plot for the data. The ‘elbow’ of the graph appears to indicate retaining three factors. Parallel analysis indicated that two factors occurred above chance based on the 95th percentile. However, the difference between the third‐factor eigenvalue for the data set and that produced for the random eigenvalue within the parallel analysis was small (difference of 0.111). Based on the theoretical underpinnings of the measure, the results of the scree plot and the parallel analysis, we examined a three‐factor solution.

With three factors extracted, a cumulative percentage of 51.09% of the total variance was explained (Factor 1, 33.36%; Factor 2, 11.92% and Factor 3, 5.82%). Direct Oblimin rotation revealed that factors 1 and 2 correlated (*r* = .426), factors 1 and 3 correlated (*r *= .480) and that factors 2 and 3 did not correlate (*r *= .060). One hypothesized avoidance item did not load on any factor above .4 and was removed (item: ‘When I'm feeling stressed, I prefer being on my own to being in the company of other people'). One hypothesized disorganized item loaded substantially on factors 1 and 3 and was also removed (item: ‘I want to be close to others but I am afraid of getting hurt’). The results of the rotated, re‐scaled factor matrix following re‐running the EFA with items removed are shown in Table 3.

**Table 3:** Extracted factors and items with item factor loadings and predicted subscale

Item	Predicted subscale	Factor		
		1	2	3
I feel frightened in close relationships	Disorganized	.796		
When I try to get close to someone sometimes I shut down and find it difficult to think or move	Disorganized	.761		
I find close relationships overwhelming	Disorganized	.739		
I often freeze when I try to get close to someone	Disorganized	.627		
I want close relationships, but being close makes me feel frightened	Disorganized	.626		
I feel uncomfortable when other people want to get to know me better	Avoidance	.586		
I want to be close to others but I often find myself pulling away when I am	Disorganized	.578		
Sometimes I am confused by my feelings towards others	Disorganized	.553		
When I form close relationships I lose sense of who I am	Disorganized	.487		
I find people I am in close relationships with to be unpredictable in their actions and behaviours	Disorganized	.439		
I usually discuss my problems and concerns with other people	Avoidance		.678	
I find it easy to depend on other people for support with problems or difficult situations	Avoidance		.665	
It helps to turn to other people when I'm stressed	Avoidance		.627	
I try to cope with stressful situations on my own	Avoidance		.536	
I find it difficult to accept help from other people when I have problems or difficulties	Avoidance		.500	
I prefer not to let other people know my ‘true’ thoughts and feelings	Avoidance		.474	
I worry that if I displease other people, they won't want to know me anymore	Anxiety			.764
I tend to get upset, anxious or angry if other people are not there when I need them	Anxiety			.740
I worry about having to cope with problems and difficult situations on my own	Anxiety			.632
When I'm stressed I want to contact close others but I am frightened of their response	Disorganized			.628
If other people disapprove of something I do, I get very upset	Anxiety			.580
I ask other people to reassure me that they care about me	Anxiety			.563
I worry a lot about my relationships with other people	Anxiety			.561
I worry that key people in my life won't be around in the future	Anxiety			.519
I worry that if other people get to know me better, they won't like me	Anxiety			.509
I often get hurt in close relationships	Disorganized			.402

Factor 1 contained the majority of items hypothesized to represent disorganized attachment, with the exception of two items which loaded with Factor 3. This factor also included one item from the original PAM avoidance subscale. The second factor consisted of six of the eight original PAM avoidance items (one item did not load on any factor above .4 and another item loaded with the disorganized items; therefore, both were removed). Factor 3 consisted of the eight original PAM anxiety subscale items plus two hypothesized disorganized items. These three factors were understood to reflect the predicted subscales disorganized, avoidance and anxiety attachment patterns respectively.

### Subgroup comparisons

Kruskal–Wallis tests indicated that there were no significant differences between scores on the revised PAM and diagnosis, gender and ethnicity. There were, however, significant differences between age range and scores on the anxiety factor. The Kruskal–Wallis test results for the anxiety factor was χ^2^(5) = 11.787, *p* = .038. Pairwise comparisons with Bonferroni correction revealed that anxiety scores were significantly higher in the 18–24 age group than the 45–4 age range; χ^2^ = 39.036, *p* = .028. No other significant differences were found between age range and scores on the revised PAM.

### Reliability

#### Internal consistency

Cronbach's alpha for the revised PAM: disorganized α = .893, avoidance α = .791 and anxiety, α = .868. Alphas for each item if deleted for all items ranged from α = .740 to α = .896, indicating that all items were relevant.

#### Test–retest reliability

ICCs (absolute agreement, two‐way mixed effects model) based on mean scores at T1 and T2 and their 95% confidence intervals (CI): disorganized ICC: = .925, 95% CI = .870–.957, *p* < .001; anxiety ICC: = .937, 95% CI = .891–.964, *p* < .001; and avoidance ICC: = .823, 95% CI = .692–.898,* p* < .001. These scores reflect excellent agreement between the T1 and T2 scores for the disorganized and anxiety factors and good agreement between the scores for the avoidance factor (Portney & Watkins, 2009), indicating measure stability.

### Construct validity of the disorganized subscale

For concurrent validity, Spearman's rank‐order correlations were explored between the disorganized factor and other measures conceptualized to assess adult disorganized attachment; the fearful subscale of the RQ and the total score of the ADA (see Table 4). These analyses revealed a large positive correlation between the disorganized factor and both the ADA and fearful category of the RQ.

**Table 4:** Spearman's rank‐order correlations between revised PAM Disorganized factor and RQ Fearful, ADA, CAPE‐42 positive frequency subscale, CAPE‐42 positive distress subscale, BBTS IT before 18 and BBTS IT after 18

	Correlation *r*_s_	Significance *p*
RQ fearful	.574	<.001
ADA	.598	<.001
CAPE positive symptoms frequency	.516	<.001
CAPE positive symptoms distress	.399	<.001
BBTS IT before 18	.398	<.001
BBTS IT after 18	.408	<.001
DES‐II total	.501	<.001

In terms of the other two subscales of the revised PAM, Spearman's rank‐order correlations indicated that there were moderate positive correlations between the anxiety factor and the RQ fearful category (*r*_s_ = .495, *p* < .001) and the ADA (*r*_s_ = .374, *p* < .001), and that there was a moderate positive correlation between the avoidance factor and the RQ fearful category (*r*_s_ = .334, *p* < .001) and a small positive correlation with the ADA (*r*_s_ = .297, *p* < .001).

For convergent validity, Spearman's rank‐order correlations were explored between the revised PAM disorganized factor and constructs hypothesized to be related conceptually to this attachment pattern (see Table 4). The revised PAM disorganized factor displayed large positive correlations with frequency of positive symptoms of psychosis (as measured by the CAPE‐42 positive symptoms subscale) and dissociation (as measured by the DES‐II). The revised PAM disorganized factor was moderately positively correlated with interpersonal trauma in childhood and adulthood (as measured by the BBTS Interpersonal Trauma items before and after 18) and distress associated with positive symptoms of psychosis (as measured by the CAPE‐42 positive symptoms subscale). The BBTS was the only scale with missing data. When the participants with missing data for the BBTS were removed, significance levels remained the same and the correlation coefficient increased slightly to *r*_s_ = .402 before the age of 18, and reduced slightly to *r*_s_ = .398 after the age of 18.

## Discussion

Disorganized attachment is an important factor in the development and maintenance of psychosis; however, this concept is not currently well captured by self‐report measures of attachment styles. The aim of this study was to expand the most well‐used measure of attachment in psychosis, the PAM (Berry et al., 2008), to capture the concept of adult disorganized attachment within a clinical sample of individuals with a self‐reported diagnosis of psychosis. Following an iterative process of development, 12 items with good content and face validity were included with the original items of the PAM to form a revised measure of 28 items in total and were administered to a large online sample. Based on our analysis and in line with theory, a three‐factor solution appears to reflect the structural dimensionality of the revised measure. This three‐factor solution reflected three subscales of disorganized, avoidant and anxious attachment. Subscales were internally consistent, reliable over time and the disorganized subscale correlated with other measures of adult disorganized attachment as well as key constructs, in line with predictions.

The disorganized factor emerged as the first factor, explaining the largest proportion of the variance and displaying large positive correlations with other measures capturing aspects of adult disorganized attachment, the ADA and fearful category of the RQ, demonstrating that the disorganized factor has good concurrent validity. These results further demonstrate that the disorganized subscale was not redundant with these other measures; that is, correlations were large and significant but did not correlate perfectly, highlighting that although related, the disorganized subscale of the revised PAM is capturing something slightly different from these measures. The fearful category of the RQ captures approach‐avoidance behaviours (Bartholomew & Horowitz, 1991), and the ADA focuses on fear of attachment figures in romantic relationships (Paetzold *et al.*, 2015). The development of the revised PAM disorganized items was informed by the concepts included in the RQ and ADA, with the exception of their focus on romantic relationships; however, the development of the new items was additionally informed by the AAI's (George *et al.*, 1996) conceptualization of unresolved attachment, following a review of representative AAI transcripts featuring narratives consistent with disorganized attachment. Therefore, we argue that the new disorganized subscale goes beyond these two existing measures, which explains why larger correlation coefficients were not established.

Research has highlighted an association between disorganized attachment and dissociation (Liotti, 2004; Longden *et al.*, 2011). In line with these findings, the disorganized subscale displayed a large positive correlation with the DES‐II total score. A large positive correlation was also shown between the new disorganized subscale and frequency of positive symptoms of psychosis, and a moderate positive correlation with distress associated with positive symptoms of psychosis, as measured by the CAPE‐42 positive symptoms subscale. These findings demonstrate a link between positive symptoms and disorganized attachment, in line with reports in the literature that disorganized attachment is over‐represented in people experiencing psychosis (around one third of individuals; Harder, 2014) and recent research conducted by Bucci *et al.* (2017), who identified that disorganized attachment is significantly associated with positive symptoms of psychosis. Again, consistent with the literature of the association between trauma and disorganized attachment (Liotti, 2004), the disorganized factor was moderately correlated with interpersonal trauma before and after the age of 18 as measured by the BBTS interpersonal trauma items. Overall, these results reflect good construct validity of the disorganized factor developed in this study.

The avoidance subscale which emerged differed from its original factor structure (Berry et al., 2008). One of the items loaded with the new disorganized items and one did not load at all above cut‐off. Differences between the avoidance subscale in this study and the original EFA may reflect the results of other studies which have failed to replicate this subscale's factor structure (Olbert *et al.*, 2016). The item which loaded with the disorganized items loaded substantially (factor loading: .586) and as such strongly indicates that it should be retained with this factor as an item capturing disorganized attachment. This study highlighted that six of the original items formed a reliable factor, demonstrating good internal consistency and stability over time, and should be retained as the avoidance subscale going forward. The EFA suggests that the item which did not load above cut‐off should not be retained in the subscale as it indicates that it is not sufficiently reflecting avoidant attachment.

The anxiety subscale which emerged from the solution retained the items from the original anxiety subscale plus two disorganized attachment items (‘When I'm stressed I want to contact close others but I am frightened of their response’ and ‘I often get hurt in close relationships’). Despite these items having established good content and face validity within the context of disorganized attachment, results from the EFA suggest that these items capture anxious attachment. It is possible that these items, although developed to reflect a fear of others in the context of attachment disorganization, may instead reflect fear and sensitivity to rejection and abandonment, which are central features of adult anxious attachment (Shaver & Mikulincer, 2002). It is therefore suggested that the items which formed this factor are retained as the anxiety subscale.

### Strengths and limitations

This study benefited from sufficient sample size with a clinical sample to allow EFA within the targeted population (individuals with psychosis) for the measure. Although 62% of the sample fell between the ages of 18 and 34, the majority were women, white British with degree level education. Additionally, the study was reliant on a self‐reported diagnosis of or treatment for a psychosis‐related difficulty. Therefore, it is possible that this online sample may not be representative of a sample experiencing psychosis, who are currently accessing or engaged with services. Similarly, there may have been people who experience psychosis but did not deem themselves eligible to take part in the study because they have neither been diagnosed with a psychosis‐related difficulty nor received treatment. Furthermore, web‐based surveys are thought to involve coverage bias, which is a biased sample due to individuals in the target population not having or choosing not to access the internet which may have occurred in the sample. Additionally, non‐response bias is a problem with online studies and may have also impacted the sample, this involves systematic differences between those who did and did not complete the survey (Morgado, Meireles, Neves, Amaral, & Ferreira, 2017). Thus, the online sampling methodology is a potential limitation of this study and further research is required to establish the generalizability of the findings and how they relate to the broader sample of people with experiences of psychosis within and outside of mental health services.

### Future research and clinical implications

This study presented EFA of the revised PAM. Further research conducting a CFA within a new sample is required (with a minimum of 130 participants to allow CFA), to confirm the factor solution. Due to limitations regarding the generalizability of the findings of the current research because of the recruitment method, individuals should be recruited face to face.

It has been emphasized that within assessment and formulation, individuals who experience psychosis should be asked about their attachment relationships (Berry & Drake, 2010). Following confirmatory psychometric validation, the revised PAM has the potential to aid clinicians in identifying and understanding the attachment pattern of clients, with specific advantages of being able to measure disorganized attachment using a simple self‐report instrument.

Although the original PAM and the revised PAM described in this study were developed and validated to assess attachment in psychosis, disorganized attachment has been implicated in the development and maintenance of multiple mental health conditions including trauma‐related conditions (Liotti, 2004) and borderline personality disorder (Fonagy, Target, Gergely, Allen, & Bateman, 2003). Therefore, the use of the revised PAM could extend to other mental health conditions where disorganized attachment is implicated, either as a predisposing or maintaining factor, once validated within these clinical groups.

### Conclusion

In summary, the PAM was expanded to include items hypothesized to capture adult disorganized attachment. The findings demonstrated a three‐factor solution displayed good internal consistency and test–retest reliability. The disorganized factor demonstrated good construct validity through correlations with other measures of adult disorganized attachment and related constructs. However, the evidence presented in this study is exploratory. CFA of the revised PAM is required to confirm its structural dimensionality. Further research is therefore warranted which addresses the limitation of generalizability of the sample within this study. Given the significant implications that have been identified between disorganized attachment and the development and maintenance of psychosis, the use of the revised PAM, once its psychometric properties have been confirmed, will offer a simple and psychometrically robust instrument that is able to assess anxious, avoidant and disorganized attachment in clinical practice and within research settings to further research evidence in this area.

## Conflicts of interest

All other authors declare no conflict of interest

## Author contributions

Catherine Pollard (Data curation; Formal analysis; Writing – original draft) Sandra Bucci (Conceptualization; Supervision; Writing – review & editing) Angus MacBeth (Conceptualization; Supervision; Writing – review & editing) Katherine Berry, PhD DClinPsy (Conceptualization; Supervision; Writing – review & editing).

## Data Availability

The data that support the findings of this study are available from the corresponding author upon reasonable request.

## References

[bjc12249-bib-0001] Bartholomew, K. (1994). Assessment of individual differences in adult attachment. Psychological Inquiry, 5(1), 23–67. 10.1207/s15327965pli0501_2

[bjc12249-bib-0002] Bartholomew, K. , & Horowitz, L. M. (1991). Attachment styles among young adults: A test of a four‐category model. Journal of Personality and Social Psychology, 61, 226–244. 10.1037/0022-3514.61.2.226 1920064

[bjc12249-bib-0003] Bentall, R. P. , Wickham, S. , Shevlin, M. , & Varese, F. (2012). Do specific early‐life adversities lead to specific symptoms of psychosis? A study from the 2007 the Adult Psychiatric Morbidity Survey. Schizophrenia Bulletin, 38, 734–740. 10.1093/schbul/sbs049 22496540 PMC3406525

[bjc12249-bib-0004] Berry, K. , Barrowclough, C. , & Wearden, A. (2008). Attachment theory: A framework for understanding symptoms and interpersonal relationships in psychosis. Behaviour Research and Therapy, 46, 1275–1282. 10.1016/j.brat.2008.08.009 18926521

[bjc12249-bib-0005] Berry, K. , & Bucci, S. (2016). What does attachment theory tell us about working with distressing voices? Psychosis, 8(1), 60–71. 10.1080/17522439.2015.1070370

[bjc12249-bib-0006] Berry, K. , & Drake, R. (2010). Attachment theory in psychiatric rehabilitation: Informing clinical practice. Advances in Psychiatric Treatment, 16, 308–315. 10.1192/apt.bp.109.006809

[bjc12249-bib-0007] Berry, K. , Roberts, N. , Danquah, A. , & Davies, L. (2014). An exploratory study of associations between adult attachment, health service utilisation and health service costs. Psychosis, 6(4), 355–358.

[bjc12249-bib-0008] Berry, K. , Varese, F. , & Bucci, S. (2017). Cognitive attachment model of voices: Evidence base and future implications. Frontiers in Psychiatry, 8, 111 10.3389/fpsyt.2017.00111 28713292 PMC5491615

[bjc12249-bib-0009] Berry, K. , Wearden, A. , Barrowclough, C. , & Liversidge, T. (2006). Attachment styles, interpersonal relationships and psychotic phenomena in a non‐clinical student sample. Personality and Individual Differences, 41(4), 707–718. 10.1016/j.paid.2006.03.009

[bjc12249-bib-0010] Bowlby, J. (1969). Attachment. Attachment and loss: Vol. 1, Loss. New York, NY: Basic Books.

[bjc12249-bib-0011] Bowlby, J. (1988). A secure base: Clinical applications of attachment theory. London, UK: Routledge.

[bjc12249-bib-0012] Brennan, K. A. , Clark, C. L. , & Shaver, P. R. (1998). Self‐report measure‐ ment of adult attachment: An integrative overview In Simpson J. A. & Rholes W. S. (Eds.), Attachment theory and close relationships (pp. 46–76). New York, NY: Guilford.

[bjc12249-bib-0013] Bucci, S. , Emsley, R. , & Berry, K. (2017). Attachment in psychosis: A latent profile analysis of attachment styles and association with symptoms in a large psychosis cohort. Psychiatry Research, 247, 243–249. 10.1016/j.psychres.2016.11.036 27930965

[bjc12249-bib-0014] Carlson, E. B. , & Putnam, F. W. (1993). An update on the Dissociative Experiences Scale. Dissociation: Progress in the Dissociative Disorders, 6(1), 16–27.

[bjc12249-bib-0015] Costello, A. B. , & Osborne, J. W. (2005). Best practices in exploratory factor analysis: Four recommendations for getting the most from your analysis. Practical Assesment, Research & Evaluation, 10, 1–9. 10.7275/jyj1-4868

[bjc12249-bib-0016] De Wolff, M. S. , & van Ijzendoorn, M. H. (1997). Sensitivity and attachment: A meta‐analysis on parental antecedents of infant attachment. Child Development, 68, 571–591. 10.1111/j.1467-8624.1997.tb04218.x 9306636

[bjc12249-bib-0017] DePrince, A. P. , & Freyd, J. J. (2004). Forgetting trauma stimuli. Psychological Science, 15, 488–492. 10.1111/j.0956-7976.2004.00706.x 15200634

[bjc12249-bib-0018] Field, A. (2009). Discovering statistics using SPSS. London, UK: Sage publications.

[bjc12249-bib-0019] Fonagy, P. , Target, M. , Gergely, G. , Allen, J. G. , & Bateman, A. W. (2003). The developmental roots of borderline personality disorder in early attachment relationships: A theory and some evidence. Psychoanalytic Inquiry, 23, 412–459. 10.1080/07351692309349042

[bjc12249-bib-0020] George, C. , Kaplan, N. , & Main, M. (1996). Adult attachment interview.

[bjc12249-bib-0021] Goldberg, L. R. , & Freyd, J. J. (2006). Self‐reports of potentially traumatic experiences in an adult community sample: Gender differences and test‐retest stabilities of the items in a brief betrayal‐trauma survey. Journal of Trauma & Dissociation, 7, 39–63. 10.1300/J229v07n03_04 16873229

[bjc12249-bib-0022] Griffin, D. W. , & Bartholomew, K. (1994). The metaphysics of measurement: The case of adult attachment In Bartholomew K. & Perlman D. (Eds.), Advances in personal relationships, vol. 5. Attachment processes in adulthood (pp. 17–52). London, UK: Jessica Kingsley Publishers.

[bjc12249-bib-0023] Harder, S. (2014). Attachment in Schizophrenia – Implications for research, prevention, and treatment. Schizophrenia Bulletin, 40, 1189–1193. 10.1093/SCHBUL/SBU133 25232144 PMC4193730

[bjc12249-bib-0024] Hinkin, T. R. (1995). A review of scale development practices in the study of organizations. Journal of Management, 21, 967–988. 10.1177/014920639502100509

[bjc12249-bib-0025] Hinkin, T. R. (1998). A brief tutorial on the development of measures for use in survey questionnaires. Organizational Research Methods, 1(1), 104–121. 10.1177/109442819800100106

[bjc12249-bib-0026] Holtgraves, T. , & Stockdale, G. (1997). The assessment of dissociative experiences in a non‐clinical population: Reliability, validity, and factor structure of the Dissociative Experiences Scale. Personality and Individual Differences, 22, 699–706. 10.1016/S0191-8869(96)00252-8

[bjc12249-bib-0027] Hutcheson, G. D. , & Sofroniou, N. (1999). The multivariate social scientist: Introductory statistics using generalized linear models. Sage.

[bjc12249-bib-0028] Kaiser, H. F. (1974). An index of factorial simplicity. Psychometrika, 39(1), 31–36. 10.1007/BF02291575

[bjc12249-bib-0029] Liotti, G. (2004). Trauma, dissociation, and disorganized attachment: Three strands of a single braid. Psychotherapy: Theory, Research, Practice, Training, 41, 472–486. 10.1037/0033-3204.41.4.472

[bjc12249-bib-0030] Longden, E. , Madill, A. , & Waterman, M. G. (2011). Dissociation, trauma, and the role of lived experience: Toward a new conceptualization of voice hearing. Psychological Bulletin, 138, 28–76. 10.1037/a0025995 22082488

[bjc12249-bib-0031] Lynn, M. R. (1986). Determination and quantification of content validity. Nursing Research, 35, 382–386. 10.1097/00006199-198611000-00017 3640358

[bjc12249-bib-0032] Madigan, S. , Bakermans‐Kranenburg, M. J. , Van Ijzendoorn, M. H. , Moran, G. , Pederson, D. R. , & Benoit, D. (2006). Unresolved states of mind, anomalous parental behavior, and disorganized attachment: A review and meta‐analysis of a transmission gap. Attachment & Human Development, 8, 89–111. 10.1080/14616730600774458 16818417

[bjc12249-bib-0033] Main, M. , & Solomon, J. (1986). Discovery of an insecure‐disorganized/disoriented attachment pattern In Brazelton T. B. & Yogman M. W. (Eds.), Affective development in infancy (pp. 95–124). Westport, CT: Ablex Publishing.

[bjc12249-bib-0034] McGrath, J. , Saha, S. , Chant, D. , & Welham, J. (2008). Schizophrenia: A concise overview of incidence, prevalence, and mortality. Epidemiologic Reviews, 30(1), 67–76. 10.1093/epirev/mxn001 18480098

[bjc12249-bib-0035] Moreno‐Küstner, B. , Martín, C. , & Pastor, L. (2018). Prevalence of psychotic disorders and its association with methodological issues. A systematic review and meta‐analyses. PLoS ONE, 13, e0195687 10.1371/journal.pone.0195687 29649252 PMC5896987

[bjc12249-bib-0036] Morgado, F. F. , Meireles, J. F. , Neves, C. M. , Amaral, A. C. , & Ferreira, M. E. (2017). Scale development: Ten main limitations and recommendations to improve future research practices. Psicologia: Reflexão E Crítica, 30(1), 3 10.1186/s41155-016-0057-1 PMC6966966 32025957

[bjc12249-bib-0037] Olbert, C. M. , Penn, D. L. , Reise, S. P. , Horan, W. P. , Kern, R. S. , Lee, J. , & Green, M. F. (2016). Assessment of attachment in psychosis: A psychometric cause for concern. Psychiatry Research, 246, 77–83. 10.1016/j.psychres.2016.09.020 27664549

[bjc12249-bib-0038] Paetzold, R. L. , Steven Rholes, W. , & Kohn, J. L. (2015). Disorganized attachment in adulthood: Theory, measurement, and implications for romantic relationships. Review of General Psychology, 19, 146–156. 10.1037/gpr0000042

[bjc12249-bib-0039] Pearce, J. , Simpson, J. , Berry, K. , Bucci, S. , Moskowitz, A. , & Varese, F. (2017). Attachment and dissociation as mediators of the link between childhood trauma and psychotic experiences. Clinical Psychology & Psychotherapy, 24, 1304–1312. 10.1002/cpp.2100 28653442

[bjc12249-bib-0040] Penn, D. L. , Mueser, K. T. , Tarrier, N. , Gloege, A. , Cather, C. , Serrano, D. , & Otto, M. W. (2004). Supportive therapy for schizophrenia: Possible mechanisms and implications for adjunctive psychosocial treatments. Schizophrenia Bulletin, 30(1), 101–112. 10.1093/oxfordjournals.schbul.a007055 15176765

[bjc12249-bib-0041] Peterson, C. H. , Peterson, N. A. , & Powell, K. G. (2017). Cognitive interviewing for item development: Validity evidence based on content and response processes. Measurement and Evaluation in Counseling and Development, 50, 217–223. 10.1080/07481756.2017.1339564

[bjc12249-bib-0042] Pilton, M. , Varese, F. , Berry, K. , & Bucci, S. (2015). The relationship between dissociation and voices: A systematic literature review and meta‐analysis. Clinical Psychology Review, 40, 138–155. 10.1016/J.CPR.2015.06.004 26117061

[bjc12249-bib-0043] Ponizovsky, A. M. , Vitenberg, E. , Baumgarten‐Katz, I. , & Grinshpoon, A. (2013). Attachment styles and affect regulation among outpatients with schizophrenia: Relationships to symptomatology and emotional distress. Psychology and Psychotherapy: Theory, Research and Practice, 86, 164–182. 10.1111/j.2044-8341.2011.02054.x 23674467

[bjc12249-bib-0044] Portney, L. G. , & Watkins, M. P. (2009). Foundations of clinical research: Applications to practice (vol. 892). Upper Saddle River, NJ: Pearson/Prentice Hall.

[bjc12249-bib-0045] Redmond, C. , Larkin, M. , & Harrop, C. (2010). The personal meaning of romantic relationships for young people with psychosis. Clinical Child Psychology and Psychiatry, 15, 151–170. 10.1177/1359104509341447 20103564

[bjc12249-bib-0046] Shaver, P. R. , & Mikulincer, M. (2002). Attachment‐related psychodynamics. Attachment & Human Development, 4, 133–161. 10.1080/14616730210154171 12467506

[bjc12249-bib-0047] Stefanis, N. C. , Hanssen, M. , Smirnis, N. K. , Avramopoulos, D. A. , Evdokimidis, I. K. , Stefanis, C. N. , … Van Os, J. (2002). Evidence that three dimensions of psychosis have a distribution in the general population. Psychological Medicine, 32, 347–358. 10.1017/S0033291701005141 11866327

[bjc12249-bib-0048] Tabachnick, B. G. , & Fidell, L. S. (2013). Using multivariate statistics (6th ed.). Boston, MA: Allyn & Bacon/Pearson Education.

[bjc12249-bib-0049] Thornicroft, G. , Brohan, E. , Rose, D. , Sartorius, N. , & Leese, M. (2009). Articles Global pattern of experienced and anticipated discrimination against people with schizophrenia: A cross‐sectional survey. The Lancet, 373, 408–415. 10.1016/S0140 19162314

[bjc12249-bib-0050] Trémeau, F. , Antonius, D. , Malaspina, D. , Goff, D. C. , & Javitt, D. C. (2016). Loneliness in schizophrenia and its possible correlates. An exploratory study. Psychiatry Research, 246, 211–217. 10.1016/J.PSYCHRES.2016.09.043 27721059

[bjc12249-bib-0051] van IJzendoorn, M. H. (1995). Adult attachment representations, parental responsiveness, and infant attachment: A meta‐analysis on the predictive validity of the Adult Attachment Interview. Psychological Bulletin, 117, 387–403. 10.1037/0033-2909.117.3.387 7777645

[bjc12249-bib-0052] van IJzendoorn, M. H. , Schuengel, C. , & Bakermans‐Kranenburg, M. J. (1999). Disorganized attachment in early childhood: Meta‐analysis of precursors, concomitants, and sequelae. Development and Psychopathology, 11, 25 10.1080/13546805.2010.495244 16506532

[bjc12249-bib-0053] Varese, F. , Barkus, E. , & Bentall, R. P. (2011). Dissociative and metacognitive factors in hallucination‐proneness when controlling for comorbid symptoms. Cognitive Neuropsychiatry, 16, 193–217. 10.1080/13546805.2010.495244 20694861

[bjc12249-bib-0054] Varese, F. , Smeets, F. , Drukker, M. , Lieverse, R. , Lataster, T. , Viechtbauer, W. , … Bentall, R. P. (2012). Childhood adversities increase the risk of psychosis: A meta‐analysis of patient‐control, prospective‐ and cross‐sectional cohort studies. Schizophrenia Bulletin, 38, 661–671. 10.1093/schbul/sbs050 22461484 PMC3406538

[bjc12249-bib-0055] Wright, E. R. , Wright, D. E. , Perry, B. L. , & Foote‐Ardah, C. E. (2007). Stigma and the sexual isolation of people with serious mental illness. Social Problems, 54, 1533–8533. 10.1525/sp.2007.54.1.78

[bjc12249-bib-0056] Yung, A. R. , Nelson, B. , Baker, K. , Buckby, J. A. , Baksheev, G. , & Cosgrave, E. M. (2009). Psychotic‐like experiences in a community sample of adolescents: Implications for the continuum model of psychosis and prediction of schizophrenia. Australian & New Zealand Journal of Psychiatry, 43, 118–128. 10.1080/00048670802607188 19153919

